# Paradoxical low severity of COVID-19 in Prader-Willi syndrome: data from a French survey on 647 patients

**DOI:** 10.1186/s13023-021-01949-4

**Published:** 2021-07-21

**Authors:** Muriel Coupaye, Virginie Laurier, Grégoire Benvegnu, Christine Poitou, Pauline Faucher, Héléna Mosbah, Gwenaelle Diene, Graziella Pinto, Laura González Briceño, Christine Merrien, Ana Camarena Toyos, Emilie Montastier, Maithé Tauber, Fabien Mourre

**Affiliations:** 1 Assistance Publique-Hôpitaux de Paris, Centre de référence Maladies Rares (PRADORT, Syndrome de Prader-Willi et autres formes rares d’obésité avec troubles du comportement alimentaire), Service de Nutrition, Hôpital Pitié-Salpêtrière, ENDO-ERN (European Reference Network on Rare Endocrine Conditions), 47-83 boulevard de l’Hôpital, 75651 Paris Cedex 13, France; 2 Assistance Publique-Hôpitaux de Paris, Centre de référence Maladies Rares (PRADORT, Syndrome de Prader-Willi et autres formes rares d’obésité avec troubles du comportement alimentaire), Hôpital Marin d’Hendaye, ENDO-ERN (European Reference Network on Rare Endocrine Conditions), Hendaye, France; 3 Centre de référence Maladies Rares (PRADORT, Syndrome de Prader-Willi et autres formes rares d’obésité avec troubles du comportement alimentaire), Service d’Endocrinologie, Obésités, Maladies Osseuses, Génétique et Gynécologie Médicale, Hôpital des Enfants, ENDO-ERN (European Reference Network on Rare Endocrine Conditions), Toulouse, France; 4 Centre de compétences Maladies Rares à Expression Psychiatrique, Service Universitaire de Psychiatrie de l’Enfant et de l’Adolescent, Centre Hospitalier Universitaire de Toulouse, Toulouse, France; 5 INSERM, Nutrition et Obésité: Approches Systémiques «NutriOmics», Sorbonne Université, Paris, France; 6 Inserm UMR 1295 - CERPOP (Centre d’Epidémiologie et de Recherche en santé des POPulations), équipe SPHERE (Santé périnatale, pédiatrique et des adolescents: approche épidémiologique et évaluative), Université Toulouse III Paul Sabatier, Toulouse, France; 7 Assistance Publique-Hôpitaux de Paris, Service d’Endocrinologie, Gynécologie et Diabétologie pédiatrique, Hôpital Necker-Enfants Malades, ENDO-ERN (European Reference Network on Rare Endocrine Conditions), Paris, France; 8 Centre de référence Maladies Rares (PRADORT, Syndrome de Prader-Willi et autres formes rares d’obésité avec troubles du comportement alimentaire), Service d’Endocrinologie, Maladies métaboliques et Nutrition, Centre Hospitalier Universitaire de Toulouse, ENDO-ERN (European Reference Network on Rare Endocrine Conditions), Toulouse, France; 9 Institut Toulousain des Maladies Infectieuses et Inflammatoires (Infinity) INSERM UMR1291, CNRS UMR5051, Université Toulouse III, Toulouse, France

**Keywords:** Prader-Willi syndrome, COVID-19, SARS-CoV-2, Obesity, Oxytocin

## Abstract

**Background:**

Patients with Prader-Willi syndrome (PWS) often have comorbidities, especially obesity, that may constitute a risk factor for severe forms of COVID-19. We aimed to assess prevalence and medical course of SARS-CoV-2 infection in children and adults with PWS. From November 2020 to January 2021, we performed a detailed medical survey on 342 adults and 305 children with PWS followed in the French reference center.

**Results:**

We obtained responses from 288 adults (84%) and 239 children (78%). From March 2020 to January 2021, 38 adults (13.2%) and 13 children (5.4%) with PWS had SARS-CoV-2 infection**.** Mean age of adults was 34.1 ± 11.9 years and mean body mass index was 40.6 ± 12.7 kg/m^2^; 82% had obesity and 37% had diabetes**.** Only 3 children (23%) had obesity and none had diabetes. Similar to the general population, the most frequent symptoms of COVID-19 were asthenia, fever, cough, headache and shortness of breath. All patients had a favorable outcome.

**Conclusion:**

PWS itself is not a risk factor for severe COVID-19 in children and adults. On the contrary, evolution of SARS-CoV-2 infection in adults with PWS seems more favorable than expected, given their comorbidities.

## Background

Prader-Willi syndrome (PWS) is a rare genetic neurodevelopmental disorder caused by loss of expression of paternal-origin imprinted alleles on chromosome 15q11-q13. Paternal deletion is found in about 60%, maternal uniparental disomy (UPD) in 36% and imprinting defects in 4% [1]. Impaired hypothalamic development is the cause of the complex PWS phenotype: from anorexia at birth to early obesity with hormonal deficiencies and behavioral problems [2].

In contrast to the extensive literature on obesity contributing to increased risk of severe COVID-19 [3, 4], there is no published report concerning the course of COVID-19 in PWS, the most common form of syndromic obesity with an incidence of approximately 1 in 21,000 newborns [5]. In May 2020, the International Prader-Willi Syndrome Organization (IPWSO) launched a survey and the first results on June 8 showed a majority of mild infections [6]. These early data were surprising given that adults with PWS often present comorbidities which can negatively impact outcomes of COVID-19 [4]. In addition, patients with PWS have difficulties in expressing complaints and decreased fever when infection occurs, which can delay diagnosis [7]. Finally, the risk of SARS-CoV-2 infection is increased in adults with PWS because many of them live in a community structure and social distancing can be difficult in adults with intellectual and developmental disability (IDD).

We thus performed a medical survey among the patients routinely followed in the French reference center of PWS in order to assess the incidence and medical characteristics (type of signs and symptoms, severity) of the COVID-19 in patients with PWS, in addition to our participation in the IPWSO survey.

## Results

### Characteristics of adults with PWS and COVID-19

Two hundred and eighty-eight responses were obtained from the 342 families contacted (84%). Among the 288 adults, 38 (13.2%) had a confirmed COVID-19 between March 2020 and January 2021 (Table 1). Mean age was 34 years, 58% were female, 89% were Caucasian and 53% had deletion. Most patients had obesity (82%) including 55% with severe obesity. Half of the patients were living in a residential group home at the time of infection. Ten patients were hospitalized when infection occurred: one for erysipelas and 9 in Hendaye hospital (cluster).

**Table 1:** Cases of COVID-19 in French adults with Prader-Willi syndrome between March 2020 and January 2021

Clinical characteristics (n = 38)		
Age (years)	34.1 ± 11.9 [18–60]	
Gender (%)	Female: 22 (58); Male: 16 (42)	
Genetic subtype (%)	Deletion: 20 (53); UPD: 16 (42); ICD: 1 (3); unknown:1	
Body Mass Index (kg/m^2^)	40.6 ± 12.7 [21.5–72.5]	
Obesity (BMI ≥ 30 kg/m^2^) (%)	31 (82)	
Severe obesity (BMI ≥ 35 kg/m^2^) (%)	21 (55)	
Massive obesity (BMI ≥ 40 kg/m^2^) (%)	16 (42)	
Type 2 diabetes (%)	12 (32)	
Type 1 diabetes (%)	2 (5)	
Hypertension (%)	14 (37)	
Treated apnea syndrome with nocturnal CPAP (%)	13 (34)	
Respiratory and/or cardiac failure (%)	6 (16)	
Past venous thrombotic events (%)	5 (13)	
Living situation at the time of COVID-19 (%)		
Residential group home	19 (50)	
Living home with family	8 (21)	
Hospital	10 (including 9 from cluster in Hendaye Hospital)	
Treatments at the time of COVID-19 (%)		
Hormones	GH: 4 (11); Thyroxine: 16 (42); SHT: 14 (37); HC: 1 (3)	
Antidiabetic treatment	14 (37)	
Antihypertensive treatment	14 (37)	
Lipid lowering therapy	4 (11)	
Vitamin D	23 (61)	
Topiramate	9 (24)	
Antidepressants	12 (32)	
Antipsychotics	21 (55)	
Anxiolytics	9 (24)	
COVID-19 diagnosis (%)		
Positive SARS-CoV-2 RT-PCR test	29 (76)	
Positive IgG against SARS-CoV-2 after infection	9 (24)	
Plaints or clinical signs (%)	24 (63)	
Asthenia	10 (26)	
Fever	15 (39)	
Cough	7 (18)	
Shortness of breath	6 (16)	
Headache	4 (11)	
Diarrhea	3 (8)	
Anosmia	1 (3)	
No clinical sign or plaints	14 (37)	
Evolution of symptomatic patients (%)		
Admitted to hospital	12 (including 9 from cluster in Hendaye Hospital)	
Admitted to ICU	0	
Death	0	
Complete recovery	24 (100)	
Time to complete recovery (days)	6.1 ± 4.4 [1–21]	

Results are expressed as mean ± SD [range] for continuous variables and as number (percentage) for categorical variables

*BMI* body mass index, *UPD* uniparental disomy, *ICD* imprinting center defect, *GH* growth hormone, *SHT* sex hormone therapy, *HC* Hydrocortisone, *CPAP* continuous positive airway pressure, *RT-PCR* Reverse Transcriptase Polymerase Chain Reaction, *IgG* immunoglobulins G. *ICU* intensive Care Unit

About 60% of patients were on vitamin D supplementation at the time of infection. Most patients were taking psychotropic treatments including antipsychotics and antidepressants in a half and one third of the cases, respectively.

### Medical characteristics of COVID-19 in adults with PWS

Symptoms of COVID-19 were common but only one adult reported anosmia, and 37% were asymptomatic (Table 1). Three adults were hospitalized for pneumonia confirmed by computed tomography and requiring oxygen administration for all and dexamethasone for one.

The nine other hospitalized adults were part from a cluster in the PWS rehabilitation unit of Hendaye Hospital (Table 2). Like all hospitalized patients, the patient at the origin of the cluster had a negative RT-PCR 72 h before admission, but was contaminated by her caregiver during the travel to the hospital. She refused to wear a mask, which led to the contamination of 8 other patients in a unit with women only. Mean age was 38.2 years. Five patients (55%) had numerous comorbidities including massive obesity (BMI ≥ 40 kg/m^2^) for all, and type 2 diabetes, hypertension, sleep apnea syndrome, respiratory failure, lower limbs edema and past venous thrombotic events for most of them. Two patients had a pneumonia confirmed by chest X-ray and requiring oxygen administration, dexamethasone and antibiotics (Table 2). Evolution was favorable with rapid full recovery in all symptomatic patients (Table 1).

**Table 2:** Characteristics of the 9 patients of the Hendaye’s cluster

Patient	Genetic	BMI	Comorbidities	Treatments at the time of infection	Clinical signs	Treatments of COVID-19			
Age	Subtype	(kg/m^2^)				Anticoagulants	O_2_	Antibiotics	Corticosteroids
P1; 45 years	Deletion	61.7	T2D; Hypertension; sleep apnea syndrome; Respiratory and cardiac failure; Lower limbs edema; past venous thrombotic events	Thyroxine Antidiabetics Antihypertensives	Fever, asthenia, headache, cough, shortness of breath, oxygen desaturation	Enoxaparin 0.6 ml * 2/day	Yes	amoxicillin/ clavulanic acid 1 g * 3 /day	DXM 6 mg/day for 12 days
P2; 60 years	Deletion	57.9	T2D; Hypertension; sleep apnea syndrome; Respiratory failure; Lower limbs edema; past venous thrombotic events	Thyroxine Vitamin D Antidiabetics Antihypertensives	Cough, shortness of breath	Enoxaparin 0.6 ml * 2/day	Yes	amoxicillin/ clavulanic acid 1 g * 3 /day	DXM 6 mg/day for 12 days
P3; 40 years	Deletion	70.8	Hypertension; sleep apnea syndrome; Respiratory and cardiac failure; Lower limbs edema; past venous thrombotic events	Thyroxine Vitamin D Antihypertensives Anxiolytics	Cough, headache, shortness of breath	Enoxaparin 0.6 ml * 2/day	Yes	No	No
P4; 22 years	Deletion	47.7	T2D; sleep apnea syndrome; Respiratory failure; Lower limbs edema	Thyroxine Antidiabetics Topiramate Antipsychotics	Fever, asthenia, shortness of breath	Enoxaparin 0.4 ml * 2/day	No	No	No
P5; 42 years	Deletion	39.9	T2D; Hypertension; Sleep apnea syndrome; Lower limbs edema	Antidiabetics Antihypertensives Lipid lowering therapy Topiramate; antidepressants	Cough shortness of breath	Enoxaparin 0.4 ml * 2/day	No	No	No
P6; 33 years	UPD	32.0		Vitamin D Antipsychotics; mood stabilisers	Fever, cough	Enoxaparin 0.4 ml * 2/day	No	No	No
P7; 21 years	Deletion	44.0	T2D	GH; SHT; Thyroxine Antidiabetics Antipsychotics Antidepressants; Anxiolytics	Asymptomatic	Enoxaparin 0.4 ml * 2/day	No	No	No
P8; 52 years	Deletion	31.5		Vitamin D	Asymptomatic	Enoxaparin 0.4 ml * 2/day	No	No	No
P9; 29 years	Deletion	26.4		GH; SHT; Thyroxine Vitamin D	Asymptomatic	Enoxaparin 0.4 ml * 2/day	No	No	No

*UPD* Uniparental disomy, *BMI* Body Mass Index, *T2D* Type 2 diabetes, *GH* Growth Hormone, *SHT* Sex Hormone Therapy, *O2* oxygen therapy,* DXM* dexamethasone

### COVID-19 survey in children with PWS

Two hundred and thirty-nine responses were obtained from the 305 families contacted (78%). Among the 239 children, 13 (5.4%) had COVID-19 (Table 3).

**Table 3:** Cases of COVID-19 in French children with Prader-Willi syndrome between March 2020 and January 2021

Clinical characteristics (n = 13)	
Age (years)	9.6 ± 4.4 [1.9–18]
Gender (%)	Female 5 (38); Male 8 (62)
Genetic subtype (%)	Deletion: 8 (62); UPD: 5 (38)
Body Mass Index (kg/m^2^)	21.8 ± 10.9
BMI Z-score	1.3 (-2.1–7.7)
Obesity (BMI Z-score ≥ 3)	3 (23)
Type 1 or 2 diabetes	0
Treated apnea syndrome with nocturnal CPAP (%)	2 (15)
Hypertension	0
Congenital heart defect (%)	1 (8)
Respiratory and/or cardiac failure (%)	0
Living situation at the time of COVID-19 (%)	
Living home with family	13 (100)
Residential group home	0
Treatments at the time of COVID-19 (%)	
Growth hormone	13 (100)
Thyroxine	8 (62)
Hydrocortisone	1 (8)
Sex Hormone therapy	1 (8)
Vitamin D	11 (85)
Topiramate	1 (8)
Antipsychotics	1 (8)
COVID-19 diagnosis (%)	
Positive SARS-CoV-2 RT-PCR test	6 (46)
Positive IgG against SARS-CoV-2 after infection	1 (8)
Suspected diagnosis*	6 (46)
Plaints or clinical signs (%)	10 (77)
Asthenia	5 (38)
Fever	4 (31)
Cough	3 (23)
Muscle pain	1 (8)
Chest tightness	1 (8)
Headache	4 (31)
Diarrhea	2 (15)
Not eating	2 (15)
Anosmia	1 (8)
No clinical sign or plaints	3 (23)
Evolution of symptomatic patients (%)	
Hospitalization	0
Death	0
Complete recovery	9 (90)
Time to complete recovery (days)	7.7 ± 5.9 [1–20]
Partial recovery	1 (persistent asthenia)

Results are expressed as mean ± SD [range] for continuous variables and as number (percentage) for categorical variables

*BMI* body mass index, *UPD* uniparental disomy, *CPAP* continuous positive-airway pressure, *RT-PCR* Reverse Transcriptase Polymerase Chain Reaction, *IgG* immunoglobulins G

*Suspected diagnosis: reporting signs and symptoms of COVID-19 and close contact with a confirmed COVID-19 case in the 14 days prior to onset of symptoms (no RT-PCR test during infection and no assessment of antibodies against SARS-CoV-2 after infection)

Mean age was 9.6 years, 38% were female, 85% were Caucasian and 62% had deletion. Three children (23%) had obesity and two had sleep apnea syndrome. All children were living at home with family at the time of infection.

Most children (85%) were on vitamin D supplementation and only one had a psychotropic treatment (neuroleptics) at the time of infection. Three quarters of children were symptomatic but only one reported anosmia. The evolution of symptomatic cases was favorable without hospitalization and total recovery occurred in 92% of patients. One child had persistent asthenia several months after infection.

## Discussion

To our knowledge, this is the first study regarding medical characteristics of COVID-19 in patients with PWS, as reported in De Groodt et al. [9]. The two other studies concerning the COVID-19 pandemic and PWS reported behavioral and physical changes in people with PWS during the pandemic, but not medical outcomes of patients with COVID-19 [10, 11].

In France, the estimated proportion of people with SARS-CoV-2 infection, based on the fact that about 3% of patients with COVID-19 are hospitalized, had already reached 11.3% in November 2020 and up to 21.3% in the Ile-de-France region [12]. According to these estimates, adults with PWS were not more infected (13.2% of the cohort) than the French general population as of January 31, 2021. Regarding children, we do not have French data to compare, but we notice a low prevalence of COVID-19 in our cohort (5.4%).

The most important finding of our study is that we did not report any serious form of COVID-19 in adults despite many comorbidities, especially obesity and diabetes. The young age of our patients (mean of 34 years) can explain this finding since age is the main known risk factor for severe COVID-19 [4]. It is difficult to compare the severity of COVID-19 in our cohort to the French general population, including hospitalization rate for COVID-19 which is estimated at about 0.5% for people between 30 and 40 years in France [13] because 10 patients with PWS (26%) were already hospitalized (including 9 in the Hendaye hospital rehabilitation center) when infection occurred.

In contrast to our findings, several studies have reported a higher mortality due to COVID-19 in patients with Down syndrome compared to general population [14, 15]. In an international survey of 1,046 patients with a mean age to 29 years, COVID-19 severity and mortality were higher, even below 40 years, compared to the general population [15]. This poor prognosis can be explained by an immune-response dysfunction in Down syndrome, while immune function does not seem to be altered in PWS (no higher infection predisposition in our routine clinical practice). In addition, several studies have reported a higher mortality in patients with IDD compared to general population, especially in those living in residential group homes [16]. Thus, it was surprising that French adults with PWS, half of whom who were living in a residential group home, not only did not develop severe COVID-19 but also had a quick recovery.

In addition to the young age of our adults’ cohort, another hypothesis explaining the absence of severe COVID-19 in adults with PWS could be the beneficial role of oxytocin (OXT). Indeed, OXT, a neuropeptide produced in the hypothalamic paraventricular nucleus and supraoptic nucleus, is an immune-regulating neuropeptide, which has recently emerged as a candidate for treatment and prevention of COVID-19 [17–19]. OXT seems to carry special functions in immunologic defense: it suppresses neutrophil infiltration and inflammatory cytokine release, activates T-lymphocytes, and antagonizes negative effects of angiotensin II and other key pathological events of COVID-19 [19]. Through these mechanisms, OXT can block viral invasion, suppress cytokine storm, reverse lymphocytopenia, and prevent progression to severe COVID-19 [17–19]. OXT system is impaired in most patients with PWS [2] and the high plasmatic levels of OXT (possibly due to overcompensation of a brain deficit) in patients with PWS could play a protective role against COVID-19.

Other hypotheses are the potential protective effects of the drugs taken by our patients. In addition, most of our patients were treated with vitamin D which could have a protective role against COVID-19 [20, 21] even if there is no consensus on the reduction of the risk of severe evolution of COVID-19 in patients without deficit in a meta-analysis [22]. Moreover, a third of adults with PWS in our cohort were on antidepressants, that may have a beneficial effect on the course of COVID-19 [23, 24]. Finally, 10 adults with diabetes took Metformin (83% of diabetics) which reduced the mortality of hospitalized patients with COVID-19 and type 2 diabetes in several studies [25–27], but it is difficult to know if Metformin is a witness of less severe diabetes or if it has a specific effect on COVID-19 in these studies. Thus, the potential protective effects of these molecules are only hypotheses that should be interpreted with caution.

Our study has limitations. The main limitation is that we did not extend the survey to all families of patients with PWS in France because we were aiming for a medical investigation in patients followed in the reference center. Thus, the survey concerned over 600 families out of about 1000 families with PWS in France. However, patients followed in the reference center are usually the most severe [28], and we are not aware of severe COVID-19 in other French patients with PWS. The second limitation is that not all of the COVID-19 cases in children were confirmed, since children testing were not usually performed in France during this period.

## Conclusion

Despite many risk factors for severe COVID-19, such as obesity and diabetes, French adults with PWS only had mild or moderate COVID-19. The main explanation is their young age (34 years) and possibly other protective mechanisms that have yet to be elucidated. Thus, PWS itself cannot be considered as a risk factor for severe COVID-19.

## Methods

### Study rational and design

Between November 2020 and January 2021, 647 families of children and adults with genetically confirmed PWS and followed in the 3 sites of the French reference center (Paris Pitié Salpêtrière and Necker, Hendaye, and Toulouse hospitals) were contacted by telephone and/or e-mails. The PWS diagnosis was genetically confirmed before the survey using routine genetic laboratory methods. First, we performed DNA-based methylation testing to detect the absence of the paternally contributed PWS region on chromosome 15q11-q13 at locus SNRPN (Small Nuclear Ribonucleoprotein Polypeptide N). Secondly, molecular mechanism was clarified using a standard fluorescence in situ hybridization (FISH) to detect a deletion. If no deletion was found, analysis of parental and proband DNA with microsatellites was performed to confirm maternal UPD.

A confirmed COVID-19 was defined as a positive Reverse Transcriptase Polymerase Chain Reaction (RT-PCR) from a nasopharyngeal swab or the presence of blood antibodies against SARS-CoV-2 after infection. A suspected COVID-19 was defined as reporting symptoms of COVID-19 and close contact with a confirmed COVID-19 case in the 14 days prior to onset of symptoms [8].

When a case of COVID-19 was reported, a detailed medical questionnaire was filled by a clinician from the center of reference and medical records were consulted. All participants of this study (patients and caregivers) were informed and gave their oral consent for participation in this study and publication of the results.

### Statistical analysis

Data are expressed as means ± SD or numbers (%). We used descriptive statistics to report the demographic information and medical data of the patients with COVID-19.

## Data Availability

Muriel Coupaye, Virginie Laurier and Fabien Mourre have stored all the data, which are available upon reasonable request.
